# Comparison Analysis Based on Complete Chloroplast Genomes and Insights into Plastid Phylogenomic of Four *Iris* Species

**DOI:** 10.1155/2022/2194021

**Published:** 2022-07-27

**Authors:** Jing-lu Feng, Li-wei Wu, Qing Wang, Yun-jia Pan, Bao-li Li, Yu-lin Lin, Hui Yao

**Affiliations:** ^1^Institute of Medicinal Plant Development, Chinese Academy of Medical Sciences and Peking Union Medical College, Beijing 100193, China; ^2^Engineering Research Center of Chinese Medicine Resource, Ministry of Education, Beijing 100193, China

## Abstract

*Iris* species, commonly known as rainbow flowers because of their attractive flowers, are extensively grown in landscape gardens. A few species, including *Belamcanda chinensis*, the synonym of *I. domestica* and *I. tectorum*, are known for their medicinal properties. However, research on the genomes and evolutionary relationships of *Iris* species is scarce. In the current study, the complete chloroplast (CP) genomes of *I. tectorum*, *I. dichotoma*, *I. japonica*, and *I. domestica* were sequenced and compared for their identification and relationship. The CP genomes of the four *Iris* species were circular quadripartite with similar lengths, GC contents, and codon usages. A total of 113 specific genes were annotated, including the *ycf1* pseudogene in all species and *rps19* in *I. japonica* alone. All the species had mononucleotide (A/T) simple sequence repeats (SSRs) and long forward and palindromic repeats in their genomes. A comparison of the CP genomes based on mVISTA and nucleotide diversity (Pi) identified three highly variable regions (*ndhF-rpl32*, *rps15-ycf1*, and *rpl16*). Phylogenetic analysis based on the complete CP genomes concluded that *I. tectorum* is a sister of *I. japonica*, and the subgenus *Pardanthopsis* with several *I. domestica* clustered into one branch is a sister of *I. dichotoma.* These findings confirm the feasibility of superbarcodes (complete CP genomes) for *Iris* species authentication and could serve as a resource for further research on *Iris* phylogeny.

## 1. Introduction

*Iris* (L.) is a genus of flowering plants, including 300 species of the Iridaceae family classified into six subgenera (subg.) [1, 2]. These species, commonly called rainbow flowers, are found in the northern hemisphere's temperate regions and are widely used in landscape gardens because of their beautiful and colorful flowers [3]. Most *Iris* species can adapt to dry environments, such as deserts, semideserts, or rocky habitats, and a few live in mesic and wetland areas [4]. *Iris* species are also used as medicinal plants. Several pharmacological studies have shown that the rhizome extracts of *Iris* species have anticancer, anti-inflammatory, and *α*-glucosidase inhibitory effects and can reduce human infarct volume [5–7]. Few species are used to treat throat-swelling diseases [8]. The dried rhizomes of *I. tectorum* and *I. domestica*, referred to as “Chuan She Gan” and “She Gan,” respectively, are used in traditional Chinese medicine, but “She Gan” is often adulterated with the dried rhizomes of *I. dichotoma* and *I. japonica*. Therefore, identifying these four species is needed for clinical safety.

*Iris* species are characterized by fan-shaped leaves, three colorful outer perianth segments, three inner perianth segments, three petaloid stigmas with a bifid crest, and underground tuberous organs [9]. However, these species have similar leaf shapes, flower shapes, and rhizome morphological characteristics. Therefore, identification based on morphological features alone is complicated, especially during the nonflowering period. The development of *I. domestica* and *I. dichotoma* hybrids has also made species identification challenging owing to the similarities between the hybrids and female parents [10]. Molecular phylogeny combined with palynology suggested that *I. tectorum* is far away from *I. japonica* [11], which is inconsistent with classical taxonomy that shows the two species with a close relationship. *I. tectorum* is a species of section (sect.) *Lophiris* of subg. *Limniris* sect. *Lophiris* contains 13 species distributed in Eastern Asia; Dykes included this rank in sect. *Evansia* [12], but this rank was later amended by Lawrence to subsection *Evansia* [13], by Rodionenko to subg. *Crossiris* [14], and finally by Mathew to sect. *Lophiris* of subg. *Limniris* [2]. Molecular phylogeny placed *I. domestica* in subg. *Pardanthopsis* [15–17] with high support rates. Goldblatt and Mabberley confirmed that *Belamcanda chinensis* is a synonym of *Iris domestica* based on molecular, karyotype, and type specimen analyses [18]. Furthermore, karyotype analysis of *Iris* species revealed that their chromosomal genetics are abundant because of their complex origin [10, 19–23]. A few taxa of *Iris* species were identified using DNA barcodes [24–27]. Wilson [28–30] made considerable progress on molecular identification and phylogeny in *Iris* species. However, taxonomy of the *Iris* species still remains complicated [10, 11, 31, 32].

Angiosperms have a circular tetramerous chloroplast (CP) genome, consisting of a pair of inverted repeats (IRs), a small single copy (SSC) region, and a large single copy (LSC) region [33, 34]. The CP genomes serve as promising tools in identifying species and analyzing phylogeny owing to their small and simple structure, conserved sequences, and moderate nucleotide substitution rate [35–37]. Few researchers analyzed the molecular phylogenies of *Iris* based on CP or nuclear DNA fragments; however, studies based on complete CP genomes are limited. Approximately 20 complete CP genomes about *Iris* species were documented in NCBI. However, the data need to be enriched to provide detailed information on the phylogeny [26, 38–45].

The current study sequenced the complete CP genomes of *I. tectorum*, *I. dichotoma*, *I. japonica*, and *I. domestica*. The study's major objectives were to (1) characterize the complete CP genome structure and functional genes, (2) analyze the codon usage, (3) identify the SSRs and long repeats, and (4) compare the whole CP genomes of *Iris* species to screen highly variable regions. The genomes were further used to uncover the phylogeny relationship among *Iris* species. The findings will lay a foundation for classifying the species and elucidating the phylogeny in Iridaceae.

## 2. Materials and Methods

### 2.1. Sample Collection

Leaves (fresh) from *I. tectorum*, *I. dichotoma*, and *I. domestica* were collected from the Institute of Medicinal Plant Development (IMPLAD), Beijing (40°2′5″N, 116°16′14″E), and those of *I. japonica* were from the Chengdu University of Traditional Chinese Medicine, Chengdu (30°24′36″N, 103°28′48″E). The leaves were stored in a −80°C freezer, and Professor Yulin Lin identified the species. Voucher specimens were deposited in the herbarium of IMPLAD, the Chinese Academy of Medical Sciences, and the Peking Union Medical College.

### 2.2. DNA Extraction and Sequencing

Total DNA was extracted from the leaf samples by using the DNeasy Plant Mini Kit (Qiagen Co., Hilden, Germany). DNA quality was detected by agarose gel (1%) electrophoresis. The libraries (insert size average, 350 bp) were generated from total DNA and sequenced on an Illumina NovaSeq 6000 system.

### 2.3. CP Genome Assembly and Annotation

Filtered reads (low quality) from raw data were generated by Fastp version 0.23.2 [46], and clean data were assembled to generate the CP genome in GetOrganelle version 1.7.5.1 [47]. The genes were annotated using GeSeq version 2.03 [48], followed by manual correction. The genome circular map was drawn by OrganellarGenomeDRAW version 1.3.1 [49]. The whole CP genome sequences of *I. japonica* (OK448493), *I. tectorum* (MW201731), *I. dichotoma* (OK448492), and *I. domestica* (*B. chinensis*; OK448491) were submitted to NCBI.

### 2.4. Genome Structure and Codon Usage Analyses

Furthermore, MEGA X [50] was used to examine the GC content of the genome. CodonW version 1.4.2 was used to calculate the codon usage using the relative synonymous codon usage (RSCU) value as follows: there is no preference in codon usage (RSCU = 1), the codon usage frequency is less than expected (RSCU > 1), and the codon usage frequency is more than expected (RSCU < 1) [51, 52].

### 2.5. SSR and Long Repeat Sequence Analyses

The SSRs were examined by using the Microsatellite Identification tool version 2.1 [53, 54], with the parameters mentioned by Cui et al. [55]. In addition, the forward (F), palindromic (P), reverse (R), and complement (C) types of long repeat sequences with different sizes in the CP genomes were searched by using REPuter version 3.0 [56] with 30 bp as the minimum repeat size and 3 as the hamming distance.

### 2.6. Comparative Genome Analysis

The CP genomes from *I. tectorum*, *I. dichotoma*, *I. japonica*, and *I. domestica* were aligned using the mVISTA program [57]. The sequences of the shared genes in the four *Iris* species and the complete CP genomes were further aligned using MAFFT version 7 [58]. Nucleotide diversity (Pi) was calculated using DnaSP version 6 [59] to identify the divergence hotspot regions among the four species.

### 2.7. Phylogenetic Analysis

Twenty-two CP genomes of *Iris* species were downloaded from NCBI to conduct a phylogenetic tree abided by the maximum likelihood (ML) method in IQ-TREE version 2 with 1000 bootstrap replicates. *Sisyrinchium angustifolium* (NC_056184) was used as the outgroup (Table S5). The optimum model of nucleotide substitution, TVM+F+R3, determined by ModelFinder [60] in IQ-TREE [61] was used for the ML analysis.

## 3. Results and Discussion

### 3.1. CP Genomes of Four *Iris* Species

**Figure 1:**
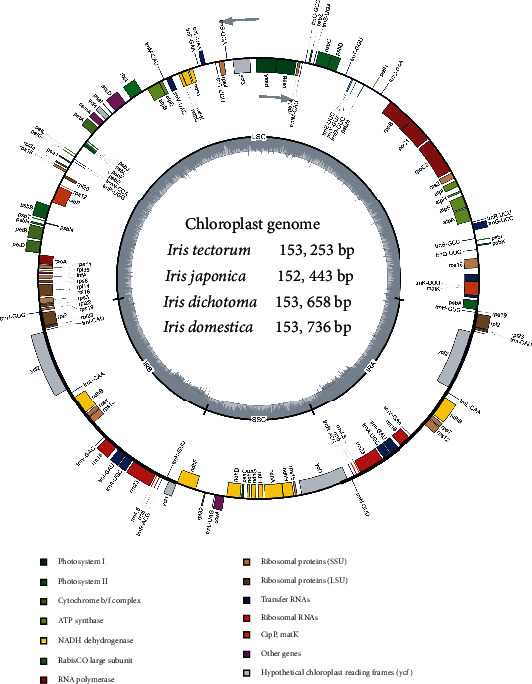
Chloroplast genome map of *Iris tectorum*. Arrows represent the transcription direction of genes. The dark (GC) and light (AT) gray areas are nucleotide contents.

Generally, sequences are chosen for molecular taxonomy, and fast (slow) molecular changes correspond to recent (old) evolution time [62]. The structure and components of the genome contribute to the nucleotide substitution rate [63, 64]. The whole CP genome is appropriate to relate species identification and relationship because of its moderate molecular changes [65]. The current study sequenced and analyzed the CP genomes of the four *Iris* species for their authentication and relationship. Illumina NovaSeq 6000 system sequencing generated 8.5, 5.3, 8.4, and 8.9 Gb of raw data for *I. tectorum*, *I. japonica*, *I. dichotoma*, and *I. domestica*, respectively. The overall lengths of the complete CP genomes were 152,443–153,736 bp as shown in Table 1. The genomes exhibited a quadripartite structure, including an SSC region (18,150–18,562 bp), an LSC region (82,833–83,237 bp), and a pair of IRs (50,716–52,428 bp; Table 1, Figure 1, and Figures S1–S3). The CP genomes of *I. tectorum*, *I. japonica*, *I. dichotoma*, and *I. domestica* had GC contents of 37.89%, 37.85%, 37.87%, and 37.85%, respectively (Table 1, Table S1) and were distributed unevenly across the four parts. The GC content illustrated in dark gray in Figure 1 was the highest in the IR region (42.97%–43.05%). This finding is probably due to the rRNA genes (*rrn4.5*, *rrn5*, *rrn16*, and *rrn23*) with less duplicated AT nucleotides [66, 67]. The LSC (35.97%–36.16%) and SSC (31.40%–31.49%) regions followed IR in terms of GC content; therefore, IR is highly conserved. Moreover, the protein-coding regions (CDS) had lengths of 78,507–79,059 bp and GC contents of 38.02%–38.15% (Table 1). The AT content at the third codon position (69.36%–69.73%) was higher than that at the second (61.75%–61.81%) and first positions (54.42%–54.48%, Table 1). These characteristics of CP genomes are different from those of nuclear and mitochondrial genomes. Moreover, these CP genome characteristics are consistent with earlier reports on *I. tectorum* [42], *I. dichotoma* [26], and *I. domestica* [26, 45]. Thus, the sequencing conducted in the current study has enriched the CP genome data of *Iris* species and could serve as an essential source for species identification and phylogeny.

A total of 113 specific genes were annotated in each CP genome, including 79 CDS genes, 30 tRNA genes, and 4 rRNA genes (Table 2). The pseudogene *ycf1* was found in all these species, whereas the pseudogene *rps19* was found only in *I. japonica*. In these species, 19 genes (18 in *I. japonica*), including 7 (6 in *I. japonica*) CDS genes, 8 tRNA genes, and 4 rRNA genes, were repeated twice in IRs. Moreover, 15 genes, including 9 CDS and 6 tRNA genes, contained 1 intron, whereas 3 genes contained 2 introns (Table 2). The CDS lengths of *I. tectorum*, *I. japonica*, *I. dichotoma*, and *I. domestica* were 78,957, 78,507, 79,050, and 79,059 bp, respectively, and accounted for 51.52%, 51.50%, 51.45%, and 51.43% of the genome, respectively. In *I. tectorum*, the rRNAs were 9,050 bp long (5.91%), and the tRNAs were 2,878 bp long (1.88%). The lengths and proportions of rRNAs and tRNAs in *I. japonica*, *I. dichotoma*, and *I. domestica* are shown in Table S2. In addition, the noncoding regions, including introns, intergenic spacers (IGSs), and pseudogenes, constituted 40.69%, 40.67%, 40.79%, and 40.81% of the CP genomes of *I. tectorum*, *I. japonica*, *I. dichotoma*, and *I. domestica*, respectively (Tables 1 and 2 and Table S2). These observations revealed the similarities in genomic features among these four species, indicating a close relationship.

### 3.2. Codon Usage

**Figure 2:**
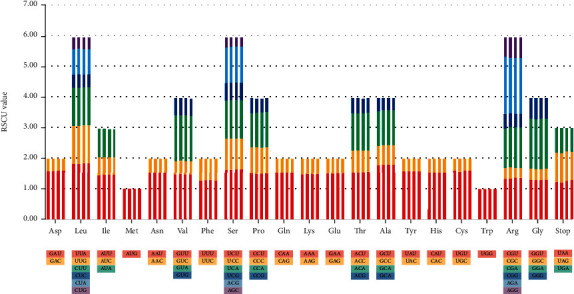
Codon usage of 20 amino acids and stop codons of the CDS in the CP genomes of *Iris* species. The four histograms from left to right in each amino acid represent *I. tectorum*, *I. japonica*, *I. dichotoma*, and *I. domestica*.

The CP genomes from *I. tectorum*, *I. japonica*, *I. dichotoma*, and *I. domestica* comprised 26,319, 26,169, 26,350, and 26,353 amino acid codons, respectively. The analysis of 64 codons encoding 20 amino acids (Figure 2 and Table S3) revealed that six codon types encoded leucine (Leu), serine (Ser), and arginine (Arg); these amino acids had maximum codons. However, one codon type encoded methionine (Met) and tryptophan (Try), and these amino acids had the least number of codons. Leucine was the most frequently coded amino acid (*I. tectorum*, 2696, 10.24%; *I. japonica*, 2661, 10.17%; *I. dichotoma*, 2692, 10.22%; and *I. domestica*, 2692, 10.22%), whereas cysteine (Cys) was the least coded (*I. tectorum*, 305, 1.16%; *I. japonica*, 303, 1.16%; *I. dichotoma*, 304, 1.15%; and *I. domestica*, 305, 1.16%).

Furthermore, the RSCU value was measured to determine nonuniform synonymous codon usage [51]. Most codons demonstrated preferences except for AUG (Met) and UGG (Try), which had RSCU values of 1. RSCU analysis revealed the presence of A or U at the third position of the preferred synonymous codons in the four *Iris* species. Other than the UGA stop codon, the CUA of leucine, and the AUA of isoleucine (Ile), the codons with A or U at the third position had RSCU values greater than 1, indicating the preferential usage of A or U. The RSCU values of the UUA of leucine were 1.84, 1.83, 1.86, and 1.86, in the CP genomes of *I. tectorum*, *I. japonica*, *I. dichotoma*, and *I. domestica*, respectively. Similarly, the RSCU values of the AGA of arginine (Arg) were 1.88, 1.83, 1.83, and 1.83, and those of the GCU of alanine (Ala) were 1.79, 1.81, 1.81, and 1.81 in *I. tectorum*, *I. japonica*, *I. dichotoma*, and *I. domestica*, respectively (Table S3). Thus, the preferential codon usage patterns were similar among these four species, which was probably due to the codon usage bias toward A/T. These similarities in codon choice also reveal the related relationship in the four species. The observed codon pattern is consistent with the CP genomes of *Amomum* [68], *Panax* [69], *Dipterygium* and *Cleome* [70], and various other species [71–73].

### 3.3. SSR and Long Repeat Sequences

**Figure 3:**
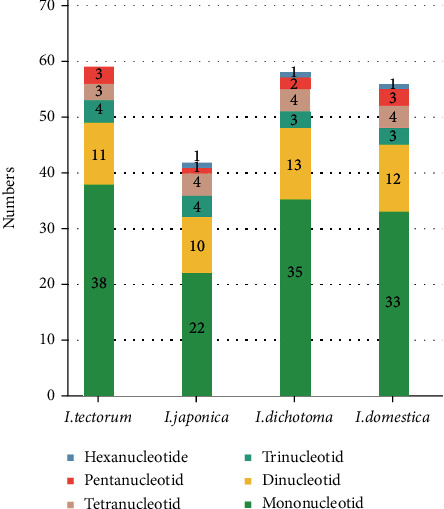
Distribution of the six types of SSRs in the CP genomes of four *Iris* species.

CP SSRs have been used as molecular markers in species authentication, population genetics, and phylogeny analysis owing to their high substitution rates [74–76]. A total of 59, 42, 58, and 56 SSRs were detected in the CP genomes of *I. tectorum*, *I. japonica*, *I. dichotoma*, and *I. domestica*, respectively (Table 3 and Table S4), including 38, 22, 35, and 33 mononucleotide SSRs; 11, 10, 13, and 12 dinucleotide SSRs; 4, 4, 3, and 3 trinucleotide SSRs; 3, 4, 4, and 4 tetranucleotide SSRs; 3, 1, 2, and 3 pentanucleotide SSRs; and 0, 1, 1, and 1 hexanucleotide SSRs, respectively (Table S4 and Figure 3). The mononucleotide repeats of *I. tectorum* and *I. japonica* had no C/G type. All four species had one AACTT/AAGTT pentanucleotide repeat. Additionally, an AAAAT/ATTTT pentanucleotide repeat was present in *I. tectorum* and *I. domestica*, whereas none was seen in *I. japonica* and *I. dichotoma*. Moreover, *I. tectorum*, *I. dichotoma*, and *I. domestica* had one specific pentanucleotide (AAAAC/GTTTT, ACTAT/AGTAT, and AATAT/ATATT, respectively). The hexanucleotide repeat (AACAAG/CTTGTT) was found in all species except *I. tectorum* (Table 3). The analysis uncovered that A/T mononucleotide repeats were mostly SSRs and account for 100.0% in *I. tectorum* and *I. japonica*, 97.1% in *I. dichotoma*, and 97.0% in *I. domestica*. Moreover, A or T base was the most frequent in the SSRs, which is similar to the base preference observed in the CP genomes of *Symplocos* [77], *Achnatherum* [78], and other species [79, 80]. These previous studies were all researched between close taxa. Therefore, the SSRs identified in this study might address the relationship among closely related *Iris* species.

**Figure 4:**
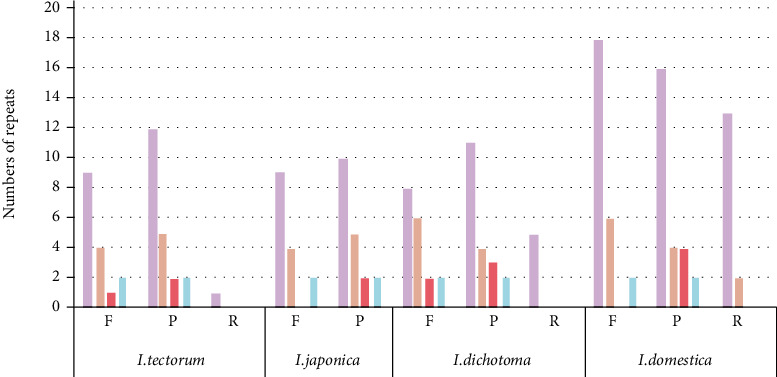
Number of long repeat sequences in the CP genomes of four *Iris* species. F, P, R, and C indicate the forward, palindromic, reverse, and complement repeats. Histograms in different colors represent repeats with different lengths.

Long repeat sequences (F, P, R, and C types) are ≥30 bp long sequences and are generally located in the IGS and intron; these repeat sequences are responsible for CP genome rearrangement and genetic diversity in populations and used as sources to uncover phylogeny relationships [81, 82]. The current study analyzed the number of long repeats within *Iris* species (Figure 4). A total of 38, 34, 43, and 67 long repeats were identified in *I. tectorum*, *I. japonica*, *I. dichotoma*, and *I. domestica*, respectively. Most of the long repeats were F and P types, accounting for 97.37% in *I. tectorum*, 100.00% in *I. japonica*, 88.37% in *I. dichotoma*, and 77.61% in *I. domestica*. The 30–39 bp long F and P types were the majority in the *Iris* species: >50% for *I. tectorum*, *I. japonica*, and *I. domestica* and 44% for *I. dichotoma*. Moreover, the repeats with ≥70 bp were all F and P types. None of the species had a C repeat, and *I. japonica* had no R repeat. In addition, *I. tectorum*, *I. dichotoma*, and *I. domestica* had 1, 5, and 15 R types, respectively. The distribution of repeats in the *Iris* species was similar to that of *Camellia* [83], *Saraca* [84], and various other species [85–87]. These repeats, one of the CP genome's various origins, are used in elucidating the phylogeny relationships of *Iris* species.

### 3.4. Inverted Repeat Expansion and Contraction

**Figure 5:**
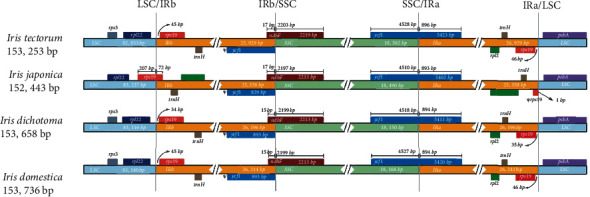
Junctions of the LSC, IR, and SSC regions in the CP genomes of the four *Iris* species. Numbers above genes indicate the distance between the gene ends and border sites. These features are not to scale. *Ψ* indicates a pseudogene.

The comparison of boundaries in the CP genomes from *I. tectorum*, *I. japonica*, *I. dichotoma*, and *I. domestica* revealed highly conserved LSC/IR/SSC conjunctional regions in the four species; however, variations were detected in the *rps19*, *ndhF*, and *ycf1* genes (Figure 5). The *rps19* gene was located 45, 34, and 45 bp away from the LSC/IRb boundary in *I. tectorum*, *I. dichotoma*, and *I. domestica*, respectively. In *I. japonica*, the *rps19* gene extended into the IRb region (72 bp), creating the *rps19* pseudogene in the IRa region. The *ndhF* gene crossed the SSC/IRb boundary in all species. Moreover, the *ycf1* gene was located in the SSC/IRa boundary, resulting in a *pseudogene* 895 bp long in *I. tectorum*, 892 bp in *I. japonica*, and 893 bp in *I. domestica* and *I. dichotoma* in the IRb region. These observations suggest that the incomplete duplications at the boundaries probably knocked down the coding potential of the *rps19* gene in *I. japonica* and the *ycf1* gene in all four *Iris* species; these expansions in IR boundaries are consistent with those in *Passiflora* [88], *Lagerstroemia* [89], and various other species [90, 91]. Divergence variations due to IR expansion among interspecies will help distinguish closely related *Iris* species.

### 3.5. Identification of Highly Variable Regions

**Figure 6:**
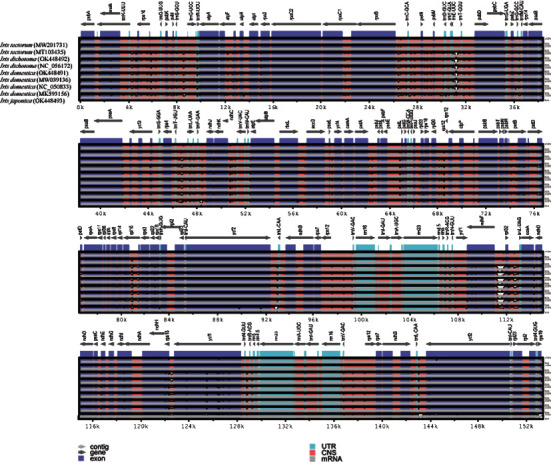
Alignment of the complete CP genomes of nine *Iris* species with the reference *I. tectorum* (MW201731) using the mVISTA program. White plots show various regions among species. The genomic regions are color coded. The vertical scale represents the percent identity ranging from 50% to 100%.

The complete CP genomes of the four *Iris* species were compared by using the mVISTA [57] program with those available sequences of *I. tectorum* (MT103435), *I. dichotoma* (NC_056172), *I. domestica* (MW039136), *I. domestica* (NC_050833), and *I. domestica* (MK593156) downloaded from GenBank. The annotated genome sequence of *I. tectorum* (MW201731) was used as the reference (Figure 6). *I. domestica* had the biggest genome (153,736 bp), and *I. japonica* had the smallest genome (152,443 bp). The reference *I. tectorum* genome (153,253 bp) was the third in size. The coding regions had less divergence than the noncoding sequence regions owing to the variable regions [92–94]. The IR regions were more conserved, whereas the LSC and SSC regions were more divergent.

**Figure 7:**
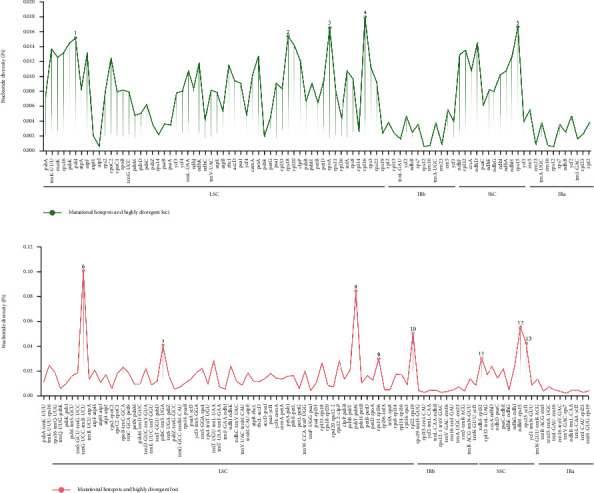
Nucleotide diversity of the gene (a) and intergenic spacer regions (b) on the whole CP genomes of four *Iris* species (Pi > 0, length > 100 bp).

Furthermore, the average Pi values [95, 96] were calculated separately for the shared genes and IGS to compare the DNA polymorphisms and identify the highly variable regions (Figure 7). The average Pi value of the gene regions was 0.00733 (Figure 7(a)), and that of the IGSs was 0.01629 (Figure 7(b)). LSC and SSC were higher than the IR regions in Pi values, similar to other plants, such as *Handroanthus* [97], *Speirantha* [98], and Combretaceae [99]. Consistent with earlier reports on other species, 13 mutational hotspots and highly divergent loci were examined in the SSC and LSC regions (Pi > 0.03 for IGS and Pi > 0.015 for gene regions), which is helpful for species authentication. The most remarkable divergent loci were *trnG-UCC-trnR-UCU* (Pi = 0.10078) and *rpl16* (Pi = 0.0178) in the IGS and gene regions, respectively. Finally, the combination of the mVISTA plots (divergent regions indicated in white) and the Pi values screened two IGSs, *ndhF-rpl32* (Figure 7(b), 11) and *rps15-ycf1* (Figure 7(b), 13), and the *rpl16* gene (Figure 7(a), 4). These regions with large white plots and high Pi values will serve as potential DNA barcodes for *Iris* species authentication.

### 3.6. Phylogenetic Analysis

**Figure 8:**
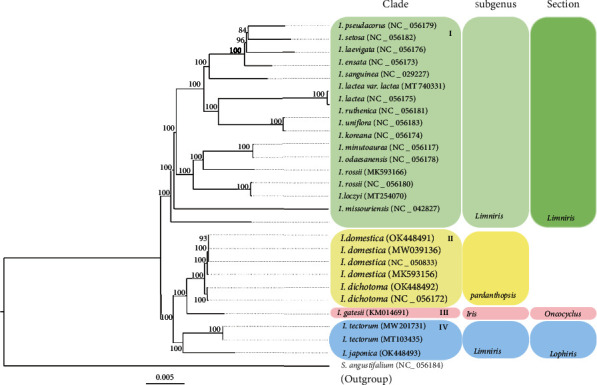
ML tree constructed based on the complete CP genomes of 26 *Iris* species and *S. angustifolium* (outgroup). Bootstrap support value is shown at each node.

CP genomes have been used to determine evolutionary relationships [100–104]. In the present study, a ML tree was constructed using 27 whole CP genome sequences to determine the evolutionary relationships of *I. tectorum*, *I. japonica*, *I. dichotoma*, and *I. domestica* with *S. angustifolium* as the outgroup (Figure 8). The phylogenetic analysis revealed the relationships between *I. tectorum* and *I. japonica* and between *I. domestica* and *I. dichotoma*. Subg. *Limniris* was divided into two clades: I (sect. *Limniris*) and IV (sect. *Lophiris*). Here, sect. *Limniris* showed a sister relationship with three clades, comprising subg. *Pardanthopsis* (clade II), subg. *Iris* (clade III), and sect. *Lophiris* (clade IV), including *I. tectorum* and *I. japonica.* These three monophyletic clades (clades I, II, and IV) were highly supported (bootstrap 100%). Moreover, subg. *Pardanthopsis* was a sister to subg. *Iris*, including *I. gatesii* of sect. *Oncocyclus* (bootstrap value of 100%); *I. domestica* and *I. dichotoma* in clade II were closely related sister species. Additionally, *I. domestica* (OK448491, *B. chinensis*) was clustered with the other three *I. domestica* sequences. This finding was consistent with the findings of Goldblatt and Mabberley [18], Mavrodiev et al. [105], and Wilson [28] who indicated that *B. chinensis* is a synonym of *I. domestica*. In addition, two *I. dichotoma* sequences (previous and present) were clustered into a branch, similar to the two sequences of *I. tectorum*. These results mutually corroborated the accuracy of the sequences. Notably, the four species were separated into distinct groups. Thus, for the first time, the present study deduced the relationship among the four *Iris* species based on complete CP genomes following the ML method. These results are consistent with the molecular phylogeny by Wilson [28], Guo and Wilson [11], Kang et al. [26], and Xiao et al. [106] based on different plastid fragments. Thus, the phylogenetic analysis uncovers that the CP genomes could be used to verify the subdivisions of *Iris* species, especially at the subgenus and section ranks.

The ML tree based on common protein-coding sequences (Figure S4) was similar to that based on the complete CP genomes (Figure 8), except for two branches, i.e., branch of *I. pseudacorus*, *I. setosa*, *I. laevigata*, and *I. ensata* species and branch of *I. domestica* and *I. dichotoma* species. In detain, *I. ensata*, in both trees, was the most primitive taxon among four species, but the *I. pseudacorus*, *I. setosa*, and *I. laevigata* demonstrated different relationships in these two trees. Meanwhile, *I. domestica* could be distinguished from *I. dichotoma* in the tree based on the complete chloroplast genomes, but the tree based on common protein-coding sequences could not differentiate *I. domestica* from *I. dichotoma*. The complete chloroplast genome has been commonly used as superbarcoding for species identification in researches, such as *Dipterygium* and *Cleome* [70] and *Zantedeschia* [91]. In the present study, the result of species authentication based on complete CP genomes among four medicinal *Iris* species also proved the efficacy of superbarcoding. The usage of complete CP genomes was more efficient than the usage of common protein-coding sequences for *Iris* species identification, probably derived from more variant regions contained in intergenic regions of the complete chloroplast genome [98, 104].

## 4. Conclusions

The present research sequenced and analyzed the complete CP genomes of four *Iris* species, namely, *I. tectorum*, *I. dichotoma*, *I. japonica*, and *I. domestica*. CP genome sizes, GC contents, codon usages, SSRs, and long repeats were examined, and the genome conservation and differences among the four *Iris* species were compared. Furthermore, comparing these species' genomes with other Iridaceae species revealed a few variable regions; however, the use of these markers in DNA barcoding needs to be tested. The study also generated an ML phylogenetic tree that depicted the evolutionary relationship of *Iris* species and confirmed that *B. chinensis* is a synonym of *I. domestica*; however, the whole CP genomes of the 13 taxa of sect. *Lophiris* need to be included in one robust phylogenetic analysis. The study's findings confirm that CP genomes are a worthy genetic resource for identifying Iridaceae species and analyzing their phylogeny.

## Figures and Tables

**Table 1:** Length and composition of the CP genomes of *I. tectorum*, *I. japonica*, *I. dichotoma*, and *I. domestica.*

Types/species	*I. tectorum*	*I. japonica*	*I. dichotoma*	*I. domestica*
Accession number	MW201731	OK448493	OK448492	OK448491
Total length (bp)	153,253	152,443	153,658	153,736
SSC (bp)	18,562	18,490	18,150	18,168
LSC (bp)	82,833	83,237	83,116	83,140
IRs (bp)	51,858	50,716	52,392	52,428
CDS (bp)	78,957	78,507	79,050	79,059
Total GC (%)	37.89	37.85	37.87	37.85
GC of SSC (%)	31.42	31.40	31.49	31.46
GC of LSC (%)	36.16	36.13	36.00	35.97
GC of IRa (%)	42.97	43.03	43.04	43.05
GC of IRb (%)	42.97	43.03	43.04	43.05
GC of CDS (%)	38.15	38.08	38.03	38.02
AT at the 1^st^ position (%)	54.42	54.48	54.43	54.44
AT at the 2^nd^ position (%)	61.77	61.81	61.75	61.77
AT at the 3^rd^ position (%)	69.36	69.46	69.73	69.72

**Table 2:** Genes in the CP genomes of *I. tectorum*, *I. japonica*, *I. dichotoma*, and *I. domestica.*

Functional group	Genes	Number of genes
Photosystem I	*psaA*, *psaB*, *psaC*, *psaI*, *psaJ*	5
Photosystem II	*psbA*, *psbB*, *psbC*, *psbD*, *psbE*, *psbF*, *psbH*, *psbI*, *psbJ*, *psbK*, *psbL*, *psbM*, *psbN*, *psbT*, *psbZ*	15
Cytochrome b/f complex	*petA*, *petB*^∗^, *petD*^∗^, *petG*, *petL*, *petN*	6
ATP synthase	*atpA*, *atpB*, *atpE*, *atpF*^∗^, *atpH*, *atpI*	6
NADH dehydrogenase	*ndhA* ^∗^, *ndhB*^∗^ (×2), *ndhC*, *ndhD*, *ndhE*, *ndhF*, *ndhG*, *ndhH*, *ndhI*, *ndhJ*, *ndhK*	12
RubisCO large subunit	*rbcL*	1
RNA polymerase	*rpoA*, *rpoB*, *rpoC1*^∗^, *rpoC2*	4
Ribosomal proteins (SSU)	*rps2*, *rps3*, *rps4*, *rps7* (×2), *rps8*, *rps11*, *rps12*^∗∗^ (×2), *rps14*, *rps15*, *rps16*^∗^, *rps18*, *rps19^Ψ^* (×2)	15
Ribosomal proteins (LSU)	*rpl2* ^∗^ (×2), *rpl14*, *rpl16*^∗^, *rpl20*, *rpl22*, *rpl23* (×2), *rpl32*, *rpl33*, *rpl36*	11
Other genes	*accD*, *clpP*^∗∗^, *matK*, *ccsA*, *cemA*, *infA*	6
Proteins of unknown function	*ycf1^Ψ^*, *ycf2* (×2), *ycf3*^∗∗^, *ycf4*	6
Transfer RNAs	38 tRNAs (8 in the IRs (×2), 6 contain one intron)	38
Ribosomal RNAs	*rrn4.5* (×2), *rrn5* (×2), *rrn16* (×2), *rrn23* (×2)	8

×2 indicates two gene copies. ∗ and ∗∗ indicate genes that contain 1 and 2 introns, respectively. *Ψ* indicates a *pseudogene*.

**Table 3:** SSRs in the CP genomes of four *Iris* species.

SSR types	Repeat units	Number				Proportion (%)			
		①	②	③	④	①	②	③	④
Mono	A/T	38	22	34	32	100.0	100.0	97.1	97.0
	C/G	—	—	1	1	—	—	2.9	3.0
Di	AT/AT	9	8	11	10	81.8	80.0	84.6	83.3
	AG/CT	2	2	2	2	18.2	20.0	15.4	16.7
Tri	AAG/CTT	2	2	2	2	50.0	50.0	66.7	66.7
	AAT/ATT	2	2	1	1	50.0	50.0	33.3	33.3
Tetra	AAAT/ATTT	2	3	3	3	66.7	75.0	75.0	75.0
	AATG/ATTC	1	1	1	1	33.3	25.0	25.0	25.0
Penta	AACTT/AAGTT	1	1	1	1	33.3	100.0	50.0	33.3
	AAAAT/ATTTT	1	—	—	1	33.3	—	—	33.3
	AAAAC/GTTTT	1	—	—	—	33.3	—	—	—
	AATAT/ATATT	—	—	—	1	—	—	—	33.3
	ACTAT/AGTAT	—	—	1	—	—	—	50.0	—
Hexa	AACAAG/CTTGTT	—	1	1	1	—	100.0	100.0	100.0

①: *I. tectorum*; ②: *I. japonica*; ③: *I. dichotoma*; ④ *I. domestica*; —: the absence of a particular type.

## Data Availability

The data supporting the study's findings are publicly available in NCBI under the accession numbers MW201731, OK448491, OK448492, and OK448493. The associated data are available in Sequence Read Archive (SRA) under the BioSample, BioProject, and SRA numbers of *Iris tectorum* (SAMN17169715, PRJNA688136, and SRR13311445), *Iris domestica* (SAMN25087045, PRJNA798580, and SRR17692213), *Iris dichotoma* (SAMN25087046, PRJNA798580, and SRR17692212), and *Iris japonica* (SAMN25087047, PRJNA798580, and SRR17692211). The sequence data are available from https://dataview.ncbi.nlm.nih.gov/object/SRR13311445, https://dataview.ncbi.nlm.nih.gov/object/SRR17692213, https://dataview.ncbi.nlm.nih.gov/object/SRR17692212 and https://dataview.ncbi.nlm.nih.gov/object/SRR17692211. The accession numbers of others used in the present study are shown in Table S5, and these were released from NCBI.

## Abbreviations

CP: Chloroplast
CDS: Protein-coding genes
SSR: Simple sequence repeat
Pi: Nucleotide diversity
subg: Subgenera
sect: Section
SSC: Small single copy
LSC: Large single copy
IR: Inverted repeat
NCBI: National Center for Biotechnology Information
RSCU: Relative synonymous codon usage
ML: Maximum likelihood
IGS: Intergenic spacers.

## Supplementary Materials

Supplementary Materials Figure S1: CP genome map of *Iris japonica*. Figure S2: CP genome map of *Iris dichotoma*. Figure S3: CP genome map of *Iris domestica*. Figure S4: ML tree constructed based on common protein-coding genes of 26 *Iris* species and *S. angustifolium* (outgroup). Bootstrap support value is shown at each node. Table S1: gene content and gene order in the chloroplast genomes of four *Iris* species. Table S2: gene catalog of *I. tectorum*, *I. japonica*, *I. dichotoma*, and *I. domestica*. Table S3: codon usage of four *Iris* species. Table S4: simple sequence repeats in the complete chloroplast genomes of *I. tectorum*, *I. japonica*, *I. dichotoma*, and *I. domestica.* Table S5: GenBank accession numbers of the complete chloroplast genome sequences used in the phylogenetic analysis.
