# Supplementary material for: Comparison Analysis Based on Complete Chloroplast Genomes and Insights into Plastid Phylogenomic of Four Iris Species
Source: Biomed Res Int. 2022 Jul 27;2022:2194021. doi: 10.1155/2022/2194021 (PMC9348943; doi:10.1155/2022/2194021)
Supplement: Supplementary Materials — Figure S1: CP genome map of Iris japonica. Figure S2: CP genome map of Iris dichotoma. Figure S3: CP genome map of Iris domestica. Figure S4: ML tree constructed based on common protein-coding genes of 26 Iris species and S. angustifolium (outgroup). Bootstrap support value is shown at each node. Table S1: gene content and gene order in the chloroplast genomes of four Iris species. Table S2: gene catalog of I. tectorum, I. japonica, I. dichotoma, and I. domestica. Table S3: codon usage of four Iris species. Table S4: simple sequence repeats in the complete chloroplast genomes of I. tectorum, I. japonica, I. dichotoma, and I. domestica. Table S5: GenBank accession numbers of the complete chloroplast genome sequences used in the phylogenetic analysis. [file 2194021.f1.docx]

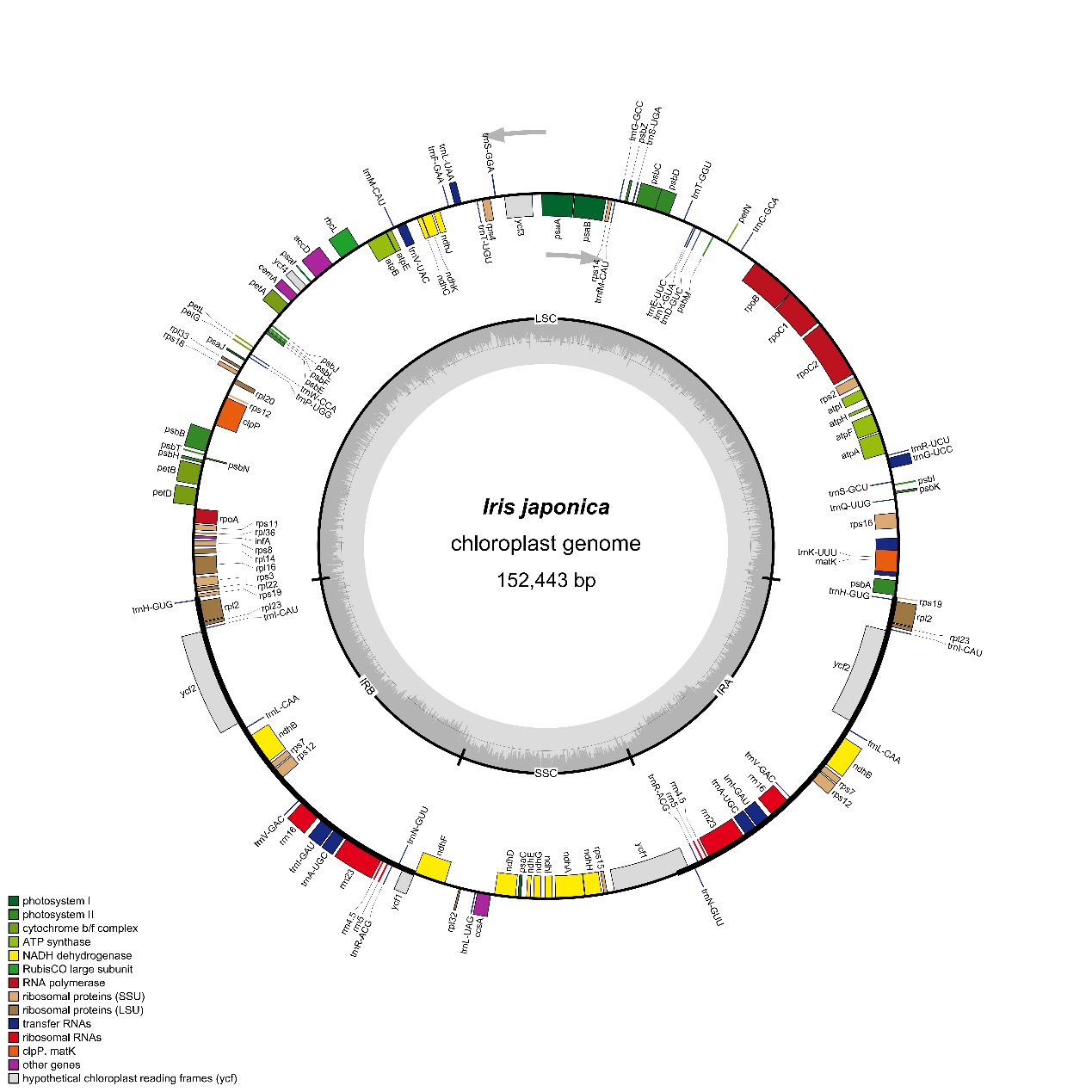


**Figure S1.** Chloroplast genome map of *Iris japonica*. Arrows represent the transcription direction of genes. The dark (GC) and light (AT) gray areas are nucleotide contents.


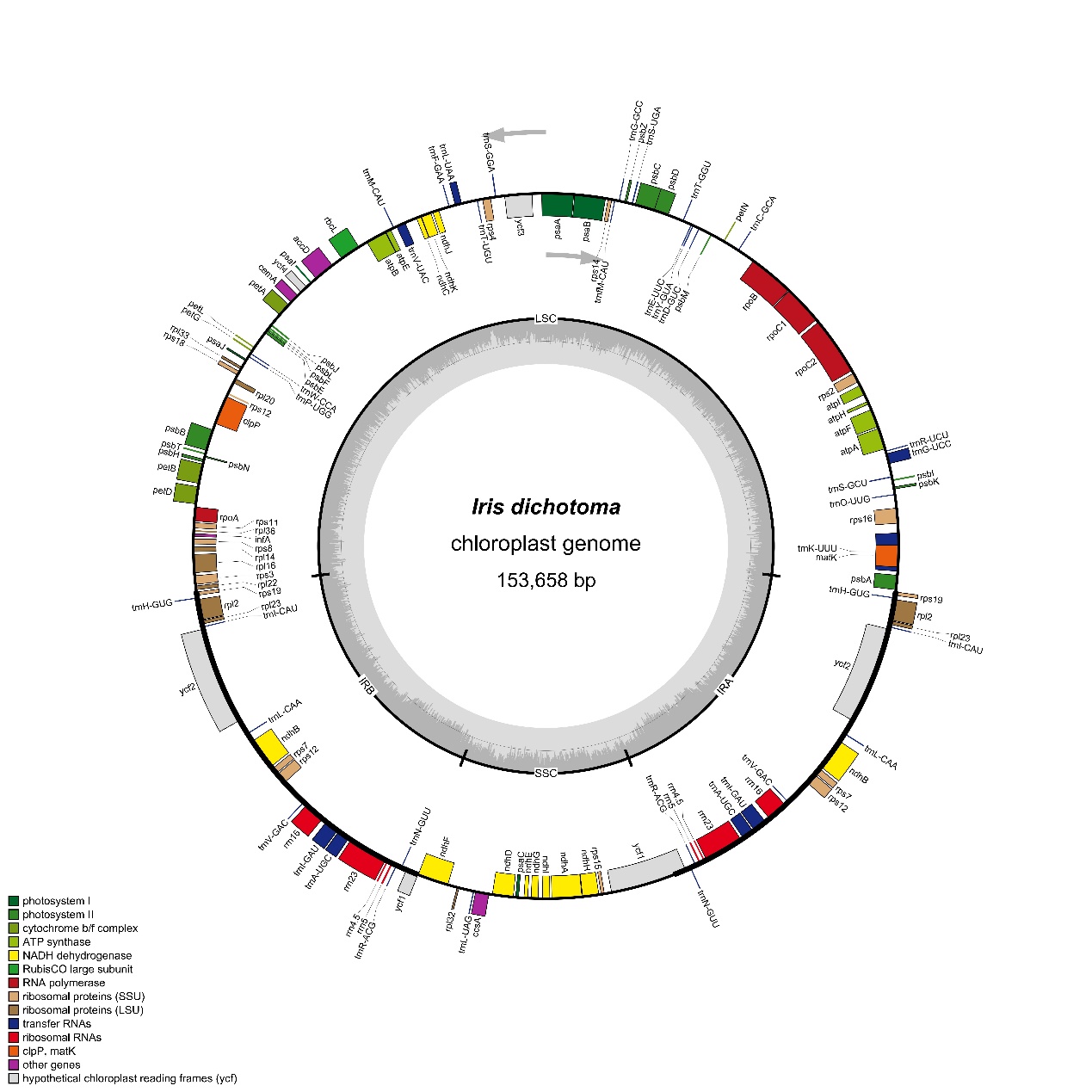


**Figure S2.** Chloroplast genome map of *Iris dichotoma*. Arrows represent the transcription direction of genes. The dark (GC) and light (AT) gray areas are nucleotide contents.


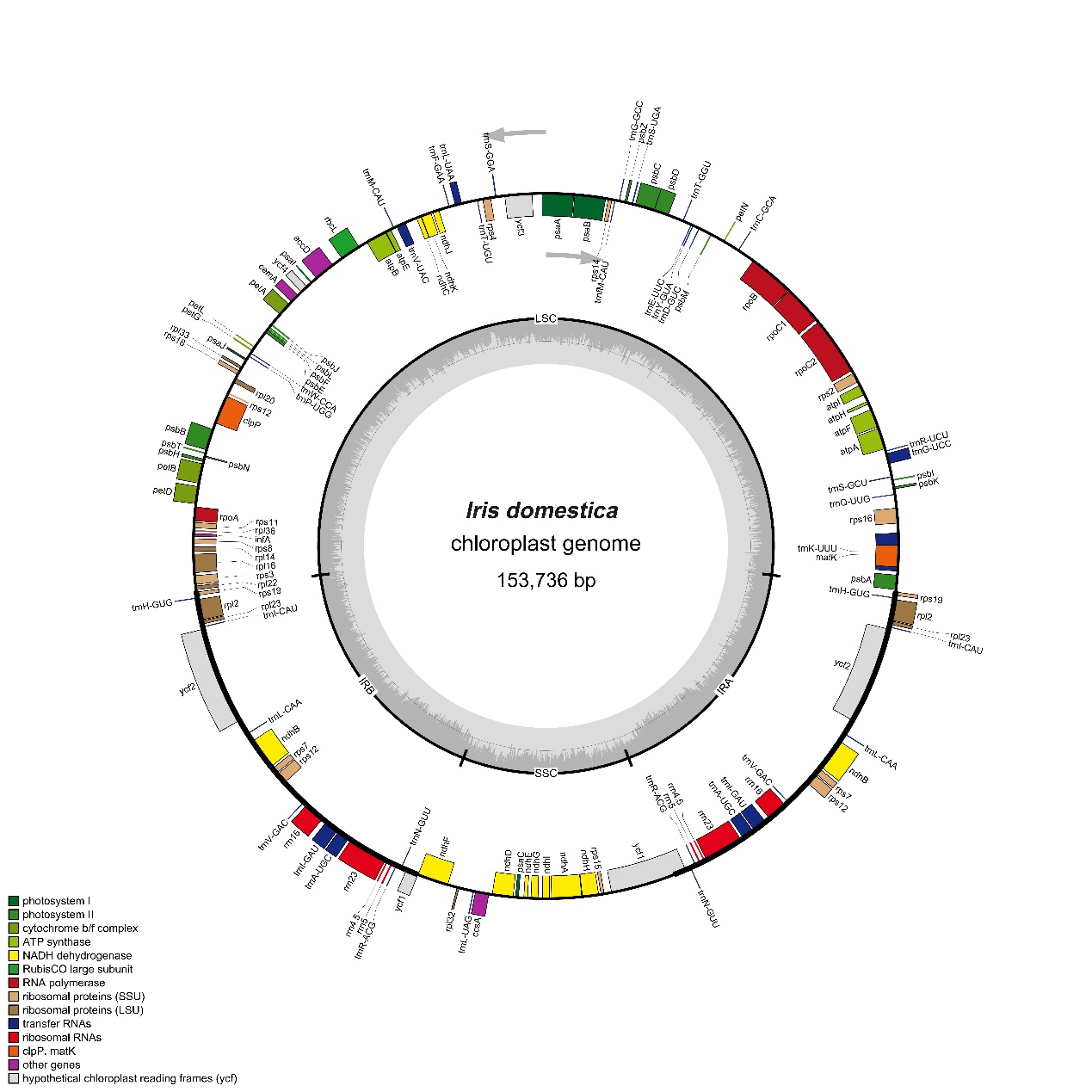


**Figure S3.** Chloroplast genome map of *Iris domestica*. Arrows represent the transcription direction of genes. The dark (GC) and light (AT) gray areas are nucleotide contents.

**Table S1.** Gene content and gene order of the chloroplast genomes of four *Irises* species.

| **Species/Types** | **Regions** | **positions** | **T(U) (%)** | **C (%)** | **A (%)** | **G (%)** | **Length (bp)** |
| --- | --- | --- | --- | --- | --- | --- | --- |
| *Iris tectorum* | IRa |  | 28.6 | 22.2 | 28.4 | 20.7 | 25,929 |
| Maximowicz | IRb |  | 28.4 | 20.7 | 28.6 | 22.2 | 25,929 |
|  | LSC |  | 32.6 | 18.5 | 31.2 | 17.6 | 82,833 |
|  | SSC |  | 34.3 | 16.6 | 34.3 | 14.8 | 18,562 |
|  | CDS |  | 31.3 | 17.8 | 30.6 | 20.4 | 78,957 |
|  |  | 1st position | 23.8 | 18.6 | 30.6 | 27.0 | 26,319 |
|  |  | 2nd position | 32.4 | 20.4 | 29.4 | 17.9 | 26,319 |
|  |  | 3rd position | 37.6 | 14.3 | 31.7 | 16.3 | 26,319 |
| *Iris japonica* | IRa |  | 28.6 | 22.3 | 28.4 | 20.7 | 25,358 |
| Thunberg | IRb |  | 28.4 | 20.7 | 28.6 | 22.3 | 25,358 |
|  | LSC |  | 32.7 | 18.6 | 31.2 | 17.6 | 83,237 |
|  | SSC |  | 34.2 | 16.7 | 34.4 | 14.7 | 18,490 |
|  | CDS |  | 31.3 | 17.7 | 30.6 | 20.3 | 78,507 |
|  |  | 1st position | 23.9 | 18.5 | 30.6 | 27.0 | 26,169 |
|  |  | 2nd position | 32.4 | 20.4 | 29.4 | 17.8 | 26,169 |
|  |  | 3rd position | 37.7 | 14.3 | 31.8 | 16.2 | 26,169 |
| *Iris dichotoma* | IRa |  | 28.5 | 22.3 | 28.4 | 20.8 | 26,196 |
| Pallas | IRb |  | 28.4 | 20.8 | 28.5 | 22.3 | 26,196 |
|  | LSC |  | 32.7 | 18.5 | 31.3 | 17.5 | 83,116 |
|  | SSC |  | 34.4 | 16.7 | 34.1 | 14.8 | 18,150 |
|  | CDS |  | 31.3 | 17.7 | 30.6 | 20.3 | 79,050 |
|  |  | 1st position | 23.8 | 18.6 | 30.6 | 27.0 | 26,350 |
|  |  | 2nd position | 32.4 | 20.4 | 29.4 | 17.8 | 26,350 |
|  |  | 3rd position | 37.8 | 14.1 | 31.9 | 16.1 | 26,350 |
| *Iris domestica* | IRa |  | 28.5 | 22.3 | 28.4 | 20.8 | 26,214 |
| (Linnaeus) | IRb |  | 28.4 | 20.8 | 28.5 | 22.3 | 26,214 |
| Goldblatt & | LSC |  | 32.7 | 18.5 | 31.3 | 17.5 | 83,140 |
| Mabberley | SSC |  | 34.4 | 16.7 | 34.2 | 14.8 | 18,168 |
|  | CDS |  | 31.3 | 17.7 | 30.6 | 20.3 | 79,059 |
|  |  | 1st position | 23.8 | 18.6 | 30.6 | 27.0 | 26,353 |
|  |  | 2nd position | 32.4 | 20.4 | 29.4 | 17.8 | 26,353 |
|  |  | 3rd position | 37.8 | 14.2 | 31.9 | 16.1 | 26,353 |

1. CDS: protein-coding regions; 2. 1st position: 1st base of codons; 3. 2nd position: 2nd

base of codons; 4. 3rd position: 3rd base of codons.

**Table S2.** Catalog of genes of *I. tectorum*, *I. japonica*, *I. dichotoma*, and *I. domestica*.

| **Species** | **Types** | | **Total** | **Numbers of genes duplicated** | **Numbers of unique genes** | **Length (bp) and %** |
| --- | --- | --- | --- | --- | --- | --- |
| *I. tectorum* |  | Protein-coding Genes (CDS) | 86 | 7 | 79 | 78,957 (51.52%) |
|  |  | rRNAs | 8 | 4 | 4 | 9,050 (5.91%) |
|  |  | tRNAs | 38 | 8 | 30 | 2,878 (1.88%) |
|  |  | Pseudogenes | 1 | 0 | 0 |  |
|  | Sub-total | Without pseudogenes | 132 | 19 | 113 |  |
|  |  | Within pseudogenes | 133 | 19 | 113 |  |
|  |  |  |  |  |  |  |
| *I. japonica* |  | Protein-coding Genes (CDS) | 85 | 6 | 79 | 78,507 (51.50%) |
|  |  | rRNAs | 8 | 4 | 4 | 9,060 (5.94%) |
|  |  | tRNAs | 38 | 8 | 30 | 2,878 (1.89%) |
|  |  | Pseudogenes | 2 | 0 | 0 |  |
|  | Sub-total | Without pseudogenes | 131 | 18 | 113 |  |
|  |  | Within pseudogenes | 133 | 18 | 113 |  |
|  |  |  |  |  |  |  |
| *I. dichotoma* |  | Protein-coding Genes (CDS) | 86 | 7 | 79 | 79,050 (51.45%) |
|  |  | rRNAs | 8 | 4 | 4 | 9,050 (5.89%) |
|  |  | tRNAs | 38 | 8 | 30 | 2,878 (1.87%) |
|  |  | Pseudogenes | 1 | 0 | 0 |  |
|  | Sub-total | Without pseudogenes | 132 | 19 | 113 |  |
|  |  | Within pseudogenes | 133 | 19 | 113 |  |
|  |  |  |  |  |  |  |
| *I. domestica* |  | Protein-coding Genes (CDS) | 86 | 7 | 79 | 79,059 (51.43%) |
|  |  | rRNAs | 8 | 4 | 4 | 9,050 (5.89%) |
|  |  | tRNAs | 38 | 8 | 30 | 2,878 (1.87%) |
|  |  | Pseudogenes | 1 | 0 | 0 |  |
|  | Sub-total | Without pseudogenes | 132 | 19 | 113 |  |
|  |  | Within pseudogenes | 133 | 19 | 113 |  |

**Table S3.** Codon usage of four *Iris* species.

| Amino Acid | Codon | Count | | | | RSCU | | | |
| --- | --- | --- | --- | --- | --- | --- | --- | --- | --- |
|  |  | ① | ② | ③ | ④ | ① | ② | ③ | ④ |
| Phe (F) | UUU | 946 | 951 | 954 | 950 | 1.25 | 1.26 | 1.26 | 1.25 |
| Phe (F) | UUC | 568 | 563 | 566 | 567 | 0.75 | 0.74 | 0.74 | 0.75 |
| Leu (L) | UUA | 828 | 812 | 834 | 835 | 1.84 | 1.83 | 1.86 | 1.86 |
| Leu (L) | UUG | 558 | 558 | 558 | 557 | 1.24 | 1.26 | 1.24 | 1.24 |
| Leu (L) | CUU | 567 | 555 | 563 | 566 | 1.26 | 1.25 | 1.25 | 1.26 |
| Leu (L) | CUC | 193 | 193 | 190 | 189 | 0.43 | 0.44 | 0.42 | 0.42 |
| Leu (L) | CUA | 374 | 371 | 373 | 372 | 0.83 | 0.84 | 0.83 | 0.83 |
| Leu (L) | CUG | 176 | 172 | 174 | 173 | 0.39 | 0.39 | 0.39 | 0.39 |
| Ile (I) | AUU | 1,073 | 1,079 | 1,084 | 1,085 | 1.43 | 1.44 | 1.44 | 1.44 |
| Ile (I) | AUC | 473 | 459 | 466 | 468 | 0.63 | 0.61 | 0.62 | 0.62 |
| Ile (I) | AUA | 705 | 708 | 701 | 702 | 0.94 | 0.95 | 0.93 | 0.93 |
| Met (M) | AUG | 634 | 638 | 639 | 639 | 1 | 1 | 1 | 1 |
| Val (V) | GUU | 517 | 518 | 520 | 519 | 1.45 | 1.46 | 1.45 | 1.45 |
| Val (V) | GUC | 169 | 169 | 168 | 169 | 0.47 | 0.48 | 0.47 | 0.47 |
| Val (V) | GUA | 537 | 530 | 539 | 536 | 1.51 | 1.49 | 1.51 | 1.5 |
| Val (V) | GUG | 204 | 204 | 204 | 205 | 0.57 | 0.57 | 0.57 | 0.57 |
| Ser (S) | UCU | 563 | 565 | 573 | 571 | 1.63 | 1.64 | 1.66 | 1.65 |
| Ser (S) | UCC | 358 | 349 | 351 | 352 | 1.04 | 1.02 | 1.02 | 1.02 |
| Ser (S) | UCA | 428 | 437 | 436 | 436 | 1.24 | 1.27 | 1.26 | 1.26 |
| Ser (S) | UCG | 201 | 195 | 197 | 197 | 0.58 | 0.57 | 0.57 | 0.57 |
| Pro (P) | CCU | 410 | 397 | 407 | 408 | 1.5 | 1.47 | 1.48 | 1.49 |
| Pro (P) | CCC | 244 | 249 | 245 | 245 | 0.89 | 0.92 | 0.89 | 0.89 |
| Pro (P) | CCA | 302 | 304 | 319 | 319 | 1.11 | 1.12 | 1.16 | 1.16 |
| Pro (P) | CCG | 136 | 131 | 127 | 126 | 0.5 | 0.48 | 0.46 | 0.46 |
| Thr (T) | ACU | 515 | 506 | 521 | 521 | 1.55 | 1.54 | 1.56 | 1.57 |
| Thr (T) | ACC | 239 | 242 | 238 | 237 | 0.72 | 0.74 | 0.71 | 0.71 |
| Thr (T) | ACA | 405 | 401 | 409 | 409 | 1.22 | 1.22 | 1.23 | 1.23 |
| Thr (T) | ACG | 168 | 163 | 164 | 164 | 0.51 | 0.5 | 0.49 | 0.49 |
| Ala (A) | GCU | 622 | 627 | 627 | 627 | 1.79 | 1.81 | 1.81 | 1.81 |
| Ala (A) | GCC | 224 | 224 | 224 | 224 | 0.64 | 0.65 | 0.65 | 0.65 |
| Ala (A) | GCA | 392 | 395 | 396 | 396 | 1.13 | 1.14 | 1.14 | 1.14 |
| Ala (A) | GCG | 155 | 142 | 142 | 142 | 0.45 | 0.41 | 0.41 | 0.41 |
| Tyr (Y) | UAU | 770 | 772 | 768 | 769 | 1.59 | 1.59 | 1.59 | 1.59 |
| Tyr (Y) | UAC | 199 | 198 | 196 | 196 | 0.41 | 0.41 | 0.41 | 0.41 |
| STOP (*) | UAA | 35 | 34 | 36 | 37 | 1.22 | 1.2 | 1.26 | 1.29 |
| STOP (*) | UAG | 28 | 28 | 28 | 27 | 0.98 | 0.99 | 0.98 | 0.94 |
| His (H) | CAU | 500 | 498 | 508 | 507 | 1.53 | 1.55 | 1.54 | 1.53 |
| His (H) | CAC | 154 | 144 | 153 | 154 | 0.47 | 0.45 | 0.46 | 0.47 |
| Gln (Q) | CAA | 692 | 700 | 700 | 698 | 1.52 | 1.52 | 1.53 | 1.53 |
| Gln (Q) | CAG | 219 | 219 | 217 | 217 | 0.48 | 0.48 | 0.47 | 0.47 |
| Asn (N) | AAU | 959 | 959 | 963 | 963 | 1.53 | 1.53 | 1.53 | 1.53 |
| Asn (N) | AAC | 294 | 298 | 297 | 297 | 0.47 | 0.47 | 0.47 | 0.47 |
| Lys (K) | AAA | 1,012 | 1,004 | 1,011 | 1,014 | 1.45 | 1.46 | 1.46 | 1.46 |
| Lys (K) | AAG | 388 | 375 | 377 | 379 | 0.55 | 0.54 | 0.54 | 0.54 |
| Asp (D) | GAU | 871 | 868 | 875 | 874 | 1.6 | 1.6 | 1.61 | 1.61 |
| Asp (D) | GAC | 217 | 217 | 215 | 215 | 0.4 | 0.4 | 0.39 | 0.39 |
| Glu (E) | GAA | 1,039 | 1,025 | 1,046 | 1,052 | 1.49 | 1.48 | 1.5 | 1.5 |
| Glu (E) | GAG | 359 | 356 | 349 | 348 | 0.51 | 0.52 | 0.5 | 0.5 |
| Cys (C) | UGU | 245 | 239 | 244 | 245 | 1.61 | 1.58 | 1.61 | 1.61 |
| Cys (C) | UGC | 60 | 64 | 60 | 60 | 0.39 | 0.42 | 0.39 | 0.39 |
| STOP (*) | UGA | 23 | 23 | 22 | 22 | 0.8 | 0.81 | 0.77 | 0.77 |
| Trp (W) | UGG | 454 | 456 | 457 | 457 | 1 | 1 | 1 | 1 |
| Arg (R) | CGU | 358 | 353 | 366 | 364 | 1.33 | 1.34 | 1.37 | 1.36 |
| Arg (R) | CGC | 96 | 100 | 88 | 89 | 0.36 | 0.38 | 0.33 | 0.33 |
| Arg (R) | CGA | 349 | 348 | 355 | 354 | 1.3 | 1.32 | 1.33 | 1.33 |
| Arg (R) | CGG | 129 | 119 | 120 | 122 | 0.48 | 0.45 | 0.45 | 0.46 |
| Ser (S) | AGU | 404 | 406 | 411 | 411 | 1.17 | 1.18 | 1.19 | 1.19 |
| Ser (S) | AGC | 113 | 110 | 106 | 106 | 0.33 | 0.32 | 0.31 | 0.31 |
| Arg (R) | AGA | 503 | 485 | 490 | 489 | 1.88 | 1.83 | 1.83 | 1.83 |
| Arg (R) | AGG | 174 | 181 | 185 | 184 | 0.65 | 0.68 | 0.69 | 0.69 |
| Gly (G) | GGU | 582 | 573 | 581 | 584 | 1.3 | 1.29 | 1.29 | 1.3 |
| Gly (G) | GGC | 168 | 164 | 165 | 164 | 0.38 | 0.37 | 0.37 | 0.37 |
| Gly (G) | GGA | 730 | 733 | 742 | 739 | 1.63 | 1.64 | 1.65 | 1.64 |
| Gly (G) | GGG | 311 | 313 | 310 | 310 | 0.69 | 0.7 | 0.69 | 0.69 |

| Amino Acid | Count subtotal | | | |
| --- | --- | --- | --- | --- |
|  | ① | ② | ③ | ④ |
| Ala (A) | 1,393 | 1,388 | 1,389 | 1,389 |
| Arg (R) | 1,609 | 1,586 | 1,604 | 1,602 |
| Asn (N) | 1,253 | 1,257 | 1,260 | 1,260 |
| Asp (D) | 1,088 | 1,085 | 1,090 | 1,089 |
| Cys (C) | 305 | 303 | 304 | 305 |
| Gln (Q) | 911 | 919 | 917 | 915 |
| Glu (E) | 1,398 | 1,381 | 1,395 | 1,400 |
| Gly (G) | 1,791 | 1,783 | 1,798 | 1,797 |
| His (H) | 654 | 642 | 661 | 661 |
| Ile (I) | 2,251 | 2,246 | 2,251 | 2,255 |
| Leu (L) | 2,696 | 2,661 | 2,692 | 2,692 |
| Lys (K) | 1,400 | 1,379 | 1,388 | 1,393 |
| Met (M) | 634 | 638 | 639 | 639 |
| Phe (F) | 1,514 | 1,514 | 1,520 | 1,517 |
| Pro (P) | 1,092 | 1,081 | 1,098 | 1,098 |
| Ser (S) | 2,067 | 2,062 | 2,074 | 2,073 |
| STOP (*) | 86 | 85 | 86 | 86 |
| Thr (T) | 1,327 | 1,312 | 1,332 | 1,331 |
| Trp (W) | 454 | 456 | 457 | 457 |
| Tyr (Y) | 969 | 970 | 964 | 965 |
| Val (V) | 1,427 | 1,421 | 1,431 | 1,429 |
| Total | 26,319 | 26,169 | 26,350 | 26,353 |
| Least (Cys) propotion | 1.16% | 1.16% | 1.15% | 1.16% |
| Most (Leu) propotion | 10.24% | 10.17% | 10.22% | 10.22% |

① *I. tectorum* ② *I. japonica* ③ *I. dichotoma* ④ *I. domestica*

**Table S4.** Simple sequence repeats (SSRs) in the complete CP genomes of *I. tectorum*, *I. japonica*, *I. dichotoma*, and *I. domestica.*

| **Species** | **SSR nr.** | **SSR type** | **SSR** | **size** | **start** | **end** |
| --- | --- | --- | --- | --- | --- | --- |
| *I. tectorum* | 1 | p1 | (T)10 | 10 | 1,606 | 1,615 |
|  | 2 | p1 | (T)12 | 12 | 2,842 | 2,853 |
|  | 3 | p5 | (TTTGT)3 | 15 | 4,541 | 4,555 |
|  | 4 | p1 | (A)10 | 10 | 4,578 | 4,587 |
|  | 5 | p1 | (A)16 | 16 | 6,305 | 6,320 |
|  | 6 | p1 | (A)11 | 11 | 7,320 | 7,330 |
|  | 7 | p1 | (T)10 | 10 | 7,576 | 7,585 |
|  | 8 | p1 | (A)10 | 10 | 9,081 | 9,090 |
|  | 9 | p1 | (A)11 | 11 | 9,717 | 9,727 |
|  | 10 | p1 | (T)10 | 10 | 9,852 | 9,861 |
|  | 11 | p2 | (TA)5 | 10 | 12,260 | 12,269 |
|  | 12 | p1 | (T)10 | 10 | 17,757 | 17,766 |
|  | 13 | p1 | (T)11 | 11 | 17,863 | 17,873 |
|  | 14 | p2 | (AT)5 | 10 | 19,230 | 19,239 |
|  | 15 | p1 | (T)10 | 10 | 21,881 | 21,890 |
|  | 16 | p1 | (T)10 | 10 | 22,279 | 22,288 |
|  | 17 | p4 | (ATAA)3 | 12 | 24,767 | 24,778 |
|  | 18 | p1 | (T)10 | 10 | 25,529 | 25,538 |
|  | 19 | p1 | (T)12 | 12 | 27,047 | 27,058 |
|  | 20 | p1 | (T)15 | 15 | 27,483 | 27,497 |
|  | 21 | p1 | (A)10 | 10 | 29,002 | 29,011 |
|  | 22 | p2 | (TA)5 | 10 | 29,170 | 29,179 |
|  | 23 | p1 | (T)12 | 12 | 29,676 | 29,687 |
|  | 24 | p2 | (AT)5 | 10 | 31,597 | 31,606 |
|  | 25 | p1 | (A)11 | 11 | 44,424 | 44,434 |
|  | 26 | p1 | (T)11 | 11 | 46,562 | 46,572 |
|  | 27 | p3 | (ATA)4 | 12 | 46,801 | 46,812 |
|  | 28 | p1 | (A)10 | 10 | 46,918 | 46,927 |
|  | 29 | p2 | (AT)5 | 10 | 46,993 | 47,002 |
|  | 30 | p2 | (AT)5 | 10 | 48,387 | 48,396 |
|  | 31 | p1 | (A)10 | 10 | 48,408 | 48,417 |
|  | 32 | p5 | (TAAGT)3 | 15 | 48,586 | 48,600 |
|  | 33 | p1 | (A)15 | 15 | 50,706 | 50,720 |
|  | 34 | p2 | (AT)6 | 12 | 58,580 | 58,591 |
|  | 35 | p4 | (AATG)3 | 12 | 60,955 | 60,966 |
|  | 36 | p1 | (A)10 | 10 | 66,927 | 66,936 |
|  | 37 | p1 | (T)10 | 10 | 69,812 | 69,821 |
|  | 38 | p1 | (T)10 | 10 | 73,047 | 73,056 |
|  | 39 | p1 | (A)11 | 11 | 74,096 | 74,106 |
|  | 40 | p1 | (T)10 | 10 | 76,878 | 76,887 |
|  | 41 | p5 | (TTTTA)3 | 15 | 78,505 | 78,519 |
|  | 42 | p1 | (T)14 | 14 | 82,387 | 82,400 |
|  | 43 | p1 | (T)10 | 10 | 82,419 | 82,428 |
|  | 44 | p2 | (GA)5 | 10 | 88,767 | 88,776 |
|  | 45 | p1 | (T)10 | 10 | 110,982 | 110,991 |
|  | 46 | p1 | (A)10 | 10 | 111,054 | 111,063 |
|  | 47 | p1 | (A)10 | 10 | 111,409 | 111,418 |
|  | 48 | p2 | (TA)5 | 10 | 111,783 | 111,792 |
|  | 49 | p4 | (AATA)3 | 12 | 114,660 | 114,671 |
|  | 50 | p1 | (A)10 | 10 | 117,377 | 117,386 |
|  | 51 | p1 | (A)10 | 10 | 118,740 | 118,749 |
|  | 52 | p1 | (A)10 | 10 | 121,999 | 122,008 |
|  | 53 | p2 | (TA)6 | 12 | 122,322 | 122,333 |
|  | 54 | p1 | (T)11 | 11 | 123,695 | 123,705 |
|  | 55 | p3 | (ATT)4 | 12 | 124,051 | 124,062 |
|  | 56 | p1 | (T)12 | 12 | 125,544 | 125,555 |
|  | 57 | p3 | (CTT)8 | 24 | 126,324 | 126,347 |
|  | 58 | p3 | (TCT)4 | 12 | 126,479 | 126,490 |
|  | 59 | p2 | (TC)5 | 10 | 147,311 | 147,320 |
| *I. japonica* | 1 | p4 | (ATAA)3 | 12 | 4,936 | 4,947 |
|  | 2 | p3 | (ATA)4 | 12 | 6,252 | 6,263 |
|  | 3 | p1 | (A)11 | 11 | 7,649 | 7,659 |
|  | 4 | p1 | (T)12 | 12 | 12,202 | 12,213 |
|  | 5 | p2 | (TA)5 | 10 | 12,248 | 12,257 |
|  | 6 | p1 | (A)10 | 10 | 13,508 | 13,517 |
|  | 7 | p1 | (A)10 | 10 | 13,954 | 13,963 |
|  | 8 | p1 | (T)11 | 11 | 15,702 | 15,712 |
|  | 9 | p1 | (A)10 | 10 | 15,931 | 15,940 |
|  | 10 | p1 | (T)11 | 11 | 17,768 | 17,778 |
|  | 11 | p1 | (T)11 | 11 | 17,875 | 17,885 |
|  | 12 | p2 | (AT)5 | 10 | 19,242 | 19,251 |
|  | 13 | p4 | (ATAA)3 | 12 | 24,787 | 24,798 |
|  | 14 | p1 | (T)10 | 10 | 25,549 | 25,558 |
|  | 15 | p1 | (A)11 | 11 | 26,425 | 26,435 |
|  | 16 | p1 | (A)10 | 10 | 26,739 | 26,748 |
|  | 17 | p1 | (T)18 | 18 | 27,711 | 27,728 |
|  | 18 | p2 | (TA)5 | 10 | 29,397 | 29,406 |
|  | 19 | p2 | (AT)5 | 10 | 32,353 | 32,362 |
|  | 20 | p1 | (T)10 | 10 | 43,293 | 43,302 |
|  | 21 | p1 | (A)10 | 10 | 44,615 | 44,624 |
|  | 22 | p2 | (AT)5 | 10 | 47,225 | 47,234 |
|  | 23 | p2 | (AT)5 | 10 | 48,612 | 48,621 |
|  | 24 | p5 | (TAAGT)3 | 15 | 48,799 | 48,813 |
|  | 25 | p2 | (AT)6 | 12 | 58,784 | 58,795 |
|  | 26 | p4 | (AATG)3 | 12 | 61,188 | 61,199 |
|  | 27 | p1 | (A)10 | 10 | 66,173 | 66,182 |
|  | 28 | p1 | (A)13 | 13 | 69,479 | 69,491 |
|  | 29 | p1 | (T)10 | 10 | 70,757 | 70,766 |
|  | 30 | p2 | (GA)5 | 10 | 88,891 | 88,900 |
|  | 31 | p1 | (A)15 | 15 | 111,242 | 111,256 |
|  | 32 | p2 | (TA)6 | 12 | 111,603 | 111,614 |
|  | 33 | p1 | (T)10 | 10 | 112,255 | 112,264 |
|  | 34 | p4 | (AATA)3 | 12 | 114,454 | 114,465 |
|  | 35 | p1 | (T)10 | 10 | 122,133 | 122,142 |
|  | 36 | p6 | (CTTGTT)3 | 18 | 123,338 | 123,355 |
|  | 37 | p1 | (T)11 | 11 | 123,477 | 123,487 |
|  | 38 | p3 | (ATT)4 | 12 | 123,833 | 123,844 |
|  | 39 | p1 | (T)12 | 12 | 125,296 | 125,307 |
|  | 40 | p3 | (CTT)6 | 18 | 126,082 | 126,099 |
|  | 41 | p3 | (TCT)5 | 15 | 126,237 | 126,251 |
|  | 42 | p2 | (TC)5 | 10 | 146,781 | 146,790 |
| *I. dichotoma* | 1 | p4 | (ATAA)3 | 12 | 4,923 | 4,934 |
|  | 2 | p1 | (A)10 | 10 | 5,085 | 5,094 |
|  | 3 | p1 | (A)12 | 12 | 7,312 | 7,323 |
|  | 4 | p1 | (A)12 | 12 | 7,643 | 7,654 |
|  | 5 | p1 | (A)10 | 10 | 9,085 | 9,094 |
|  | 6 | p1 | (T)10 | 10 | 9,704 | 9,713 |
|  | 7 | p1 | (A)12 | 12 | 9,731 | 9,742 |
|  | 8 | p1 | (T)10 | 10 | 12,217 | 12,226 |
|  | 9 | p1 | (A)13 | 13 | 12,658 | 12,670 |
|  | 10 | p1 | (A)10 | 10 | 13,955 | 13,964 |
|  | 11 | p1 | (A)10 | 10 | 15,924 | 15,933 |
|  | 12 | p1 | (T)10 | 10 | 17,762 | 17,771 |
|  | 13 | p1 | (T)11 | 11 | 17,868 | 17,878 |
|  | 14 | p2 | (AT)5 | 10 | 19,235 | 19,244 |
|  | 15 | p1 | (T)13 | 13 | 21,892 | 21,904 |
|  | 16 | p1 | (T)12 | 12 | 22,301 | 22,312 |
|  | 17 | p4 | (ATAA)3 | 12 | 24,791 | 24,802 |
|  | 18 | p1 | (T)10 | 10 | 25,553 | 25,562 |
|  | 19 | p1 | (A)11 | 11 | 26,429 | 26,439 |
|  | 20 | p1 | (T)11 | 11 | 27,743 | 27,753 |
|  | 21 | p1 | (A)10 | 10 | 29,258 | 29,267 |
|  | 22 | p2 | (TA)5 | 10 | 29,431 | 29,440 |
|  | 23 | p2 | (TA)5 | 10 | 35,684 | 35,693 |
|  | 24 | p1 | (A)12 | 12 | 44,301 | 44,312 |
|  | 25 | p2 | (TA)5 | 10 | 47,008 | 47,017 |
|  | 26 | p1 | (A)11 | 11 | 47,120 | 47,130 |
|  | 27 | p2 | (AT)5 | 10 | 47,196 | 47,205 |
|  | 28 | c* | (TA)5(A)12* | 21 | 48,653 | 48,673 |
|  | 29 | p5 | (TAAGT)3 | 15 | 48,846 | 48,860 |
|  | 30 | p1 | (T)10 | 10 | 50,957 | 50,966 |
|  | 31 | p1 | (A)11 | 11 | 50,977 | 50,987 |
|  | 32 | p1 | (A)12 | 12 | 51,252 | 51,263 |
|  | 33 | p2 | (AT)6 | 12 | 58,900 | 58,911 |
|  | 34 | p4 | (AATG)3 | 12 | 61,302 | 61,313 |
|  | 35 | p2 | (AT)5 | 10 | 67,221 | 67,230 |
|  | 36 | p1 | (T)10 | 10 | 70,135 | 70,144 |
|  | 37 | p1 | (A)11 | 11 | 70,652 | 70,662 |
|  | 38 | p4 | (TTAT)3 | 12 | 76,102 | 76,113 |
|  | 39 | p1 | (T)10 | 10 | 81,785 | 81,794 |
|  | 40 | p5 | (ACTAT)3 | 15 | 81,808 | 81,822 |
|  | 41 | p2 | (GA)5 | 10 | 89,039 | 89,048 |
|  | 42 | p1 | (T)10 | 10 | 111,866 | 111,875 |
|  | 43 | p2 | (TA)5 | 10 | 111,905 | 111,914 |
|  | 44 | p2 | (TA)5 | 10 | 111,946 | 111,955 |
|  | 45 | p2 | (AT)5 | 10 | 111,967 | 111,976 |
|  | 46 | p1 | (T)11 | 11 | 112,494 | 112,504 |
|  | 47 | p1 | (A)11 | 11 | 113,019 | 113,029 |
|  | 48 | p1 | (A)10 | 10 | 118,904 | 118,913 |
|  | 49 | p1 | (T)10 | 10 | 122,500 | 122,509 |
|  | 50 | p6 | (TTCTTG)3 | 18 | 123,705 | 123,722 |
|  | 51 | p1 | (T)11 | 11 | 123,840 | 123,850 |
|  | 52 | p3 | (ATT)4 | 12 | 124,205 | 124,216 |
|  | 53 | p1 | (C)10 | 10 | 125,499 | 125,508 |
|  | 54 | p1 | (T)12 | 12 | 125,674 | 125,685 |
|  | 55 | p3 | (CTT)7 | 21 | 126,454 | 126,474 |
|  | 56 | p3 | (TCT)7 | 21 | 126,606 | 126,626 |
|  | 57 | p2 | (TC)5 | 10 | 147,727 | 147,736 |
| *I. domestica* | 1 | p1 | (A)10 | 10 | 4,562 | 4,571 |
|  | 2 | p4 | (ATAA)3 | 12 | 4,920 | 4,931 |
|  | 3 | p1 | (A)13 | 13 | 5,082 | 5,094 |
|  | 4 | p5 | (TTATA)3 | 15 | 6,288 | 6,302 |
|  | 5 | p1 | (A)11 | 11 | 7,317 | 7,327 |
|  | 6 | p1 | (A)12 | 12 | 7,646 | 7,657 |
|  | 7 | p1 | (A)10 | 10 | 9,088 | 9,097 |
|  | 8 | p1 | (A)14 | 14 | 9,733 | 9,746 |
|  | 9 | p1 | (A)10 | 10 | 13,959 | 13,968 |
|  | 10 | p1 | (A)10 | 10 | 15,928 | 15,937 |
|  | 11 | p1 | (T)10 | 10 | 17,766 | 17,775 |
|  | 12 | p1 | (T)11 | 11 | 17,872 | 17,882 |
|  | 13 | p2 | (AT)5 | 10 | 19,239 | 19,248 |
|  | 14 | p1 | (T)11 | 11 | 21,896 | 21,906 |
|  | 15 | p1 | (T)14 | 14 | 22,303 | 22,316 |
|  | 16 | p4 | (ATAA)3 | 12 | 24,795 | 24,806 |
|  | 17 | p1 | (T)10 | 10 | 25,557 | 25,566 |
|  | 18 | p1 | (A)15 | 15 | 26,433 | 26,447 |
|  | 19 | p1 | (T)11 | 11 | 27,750 | 27,760 |
|  | 20 | p2 | (TA)5 | 10 | 29,441 | 29,450 |
|  | 21 | p1 | (T)10 | 10 | 29,947 | 29,956 |
|  | 22 | p1 | (A)10 | 10 | 31,918 | 31,927 |
|  | 23 | p2 | (TA)5 | 10 | 35,685 | 35,694 |
|  | 24 | p1 | (A)14 | 14 | 44,302 | 44,315 |
|  | 25 | p5 | (TATTT)3 | 15 | 46,881 | 46,895 |
|  | 26 | p2 | (TA)5 | 10 | 47,016 | 47,025 |
|  | 27 | p1 | (A)12 | 12 | 47,128 | 47,139 |
|  | 28 | c* | (TA)6(A)13* | 24 | 48,662 | 48,685 |
|  | 29 | p5 | (TAAGT)3 | 15 | 48,858 | 48,872 |
|  | 30 | p1 | (T)10 | 10 | 50,969 | 50,978 |
|  | 31 | p1 | (A)11 | 11 | 51,262 | 51,272 |
|  | 32 | p2 | (AT)6 | 12 | 58,887 | 58,898 |
|  | 33 | p4 | (AATG)3 | 12 | 61,294 | 61,305 |
|  | 34 | p2 | (AT)5 | 10 | 67,213 | 67,222 |
|  | 35 | p1 | (A)10 | 10 | 70,642 | 70,651 |
|  | 36 | p4 | (TTAT)3 | 12 | 76,120 | 76,131 |
|  | 37 | p1 | (T)10 | 10 | 81,178 | 81,187 |
|  | 38 | p1 | (T)13 | 13 | 81,818 | 81,830 |
|  | 39 | p1 | (T)12 | 12 | 82,724 | 82,735 |
|  | 40 | p2 | (GA)5 | 10 | 89,075 | 89,084 |
|  | 41 | p2 | (TA)6 | 12 | 111,946 | 111,957 |
|  | 42 | p2 | (TA)8 | 16 | 111,989 | 112,004 |
|  | 43 | p2 | (AT)5 | 10 | 112,016 | 112,025 |
|  | 44 | p1 | (T)11 | 11 | 112,543 | 112,553 |
|  | 45 | p1 | (A)13 | 13 | 113,058 | 113,070 |
|  | 46 | p1 | (A)10 | 10 | 118,941 | 118,950 |
|  | 47 | p1 | (T)10 | 10 | 122,814 | 122,823 |
|  | 48 | p6 | (TTCTTG)3 | 18 | 123,756 | 123,773 |
|  | 49 | p1 | (T)11 | 11 | 123,891 | 123,901 |
|  | 50 | p3 | (ATT)4 | 12 | 124,256 | 124,267 |
|  | 51 | p1 | (C)10 | 10 | 125,550 | 125,559 |
|  | 52 | p1 | (T)12 | 12 | 125,725 | 125,736 |
|  | 53 | p3 | (CTT)7 | 21 | 126,505 | 126,525 |
|  | 54 | p3 | (TCT)10 | 30 | 126,657 | 126,686 |
|  | 55 | p2 | (TC)5 | 10 | 147,793 | 147,802 |

**Table S5**. GenBank accession numbers of the complete chloroplast genome sequences used for phylogenetic analyses.

| **Species** | **GenBank acc.** | **Species** | **GenBank acc.** |
| --- | --- | --- | --- |
| *I. pseudacorus* | NC_056179 | *I. loczyi* | MT254070 |
| *I. setosa* | NC_056182 | *I. missouriensis* | NC_042827 |
| *I. laevigata* | NC_056176 | ***I. domestica*** | **OK448491** |
| *I. ensata* | NC_056173 | *I. domestica* | MW039136 |
| *I. sanguinea* | NC_029227 | *I. domestica* | NC_050833 |
| *I. lactea var. lactea* | MT740331 | *I. domestica* | MK593156 |
| *I. lactea* | NC_056175 | ***I. dichotoma*** | **OK448492** |
| *I. ruthenica* | NC_056181 | *I. dichotoma* | NC_056172 |
| *I. uniflora* | NC_056183 | *I. gatesii* | KM014691 |
| *I. koreana* | NC_056174 | ***I. tectorum*** | **MW201731** |
| *I. minutoaurea* | NC_056177 | *I. tectorum* | MT103435 |
| *I. odaesanensis* | NC_056178 | ***I. japonica*** | **OK448493** |
| *I. rossii* | MK593166 | *S. angustifolium* | NC_056184 |
| *I. rossii* | NC_056180 |  |  |


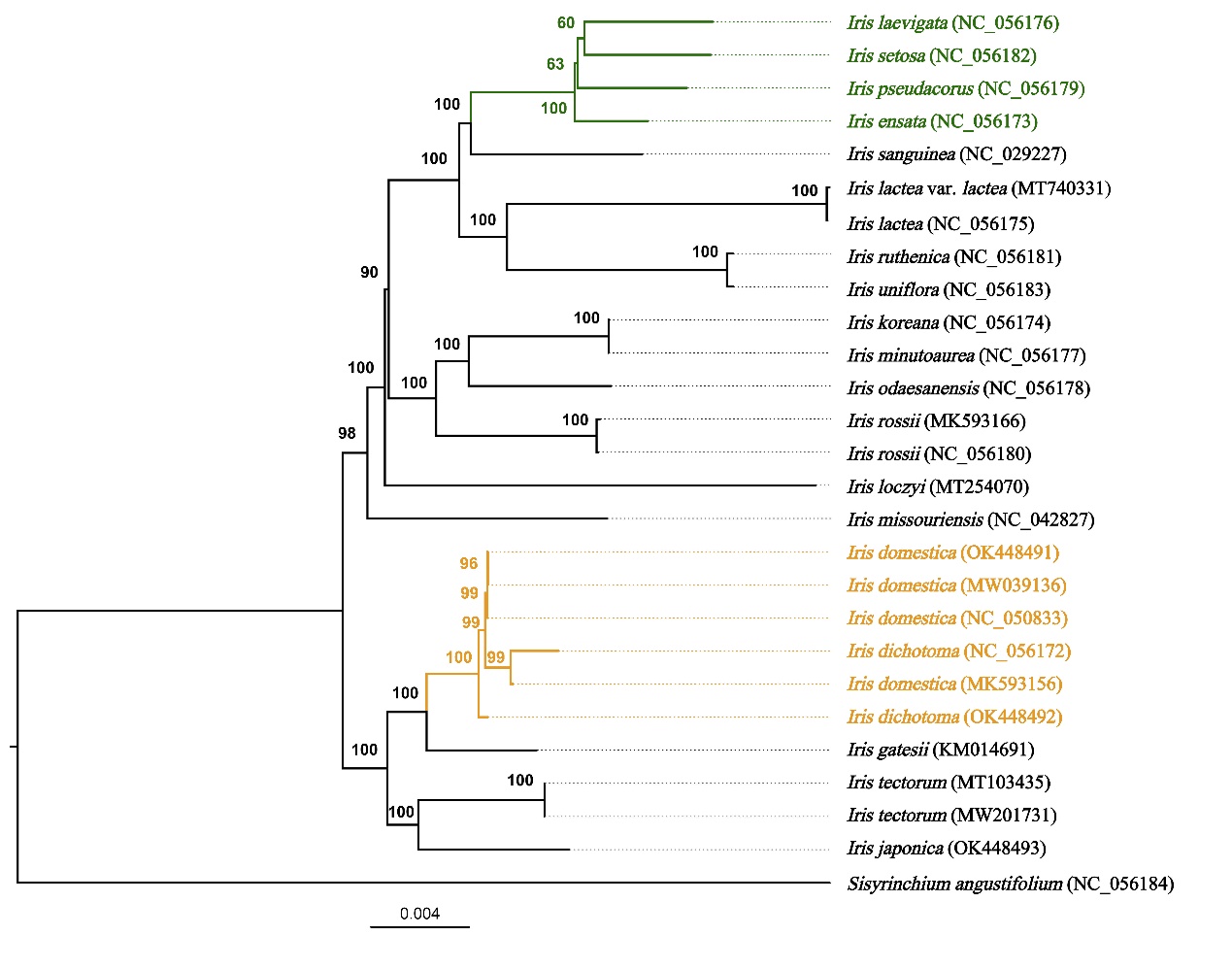


**Figure S4.** ML tree constructed based on common protein-coding genes of 26 *Iris* species and *S. angustifolium* (outgroup). Bootstrap support value is shown at each node.
